# The Role of Daily Implant-Based Multiparametric Telemonitoring in Patients with a Ventricular Assist Device

**DOI:** 10.3390/life13010038

**Published:** 2022-12-23

**Authors:** Denise Guckel, Mustapha El Hamriti, Sebastian V. Rojas, Henrik Fox, Angelika Costard-Jäckle, Jan Gummert, Thomas Fink, Vanessa Sciacca, Khuraman Isgandarova, Martin Braun, Moneeb Khalaph, Guram Imnadze, René Schramm, Michiel Morshuis, Philipp Sommer, Christian Sohns

**Affiliations:** 1 Clinic for Electrophysiology, Herz- und Diabeteszentrum NRW, Ruhr-Universität Bochum, 32545 Bad Oeynhausen, Germany; 2 Clinic for Thoracic and Cardiovascular Surgery, Herz- und Diabeteszentrum NRW, Ruhr-Universität Bochum, 32545 Bad Oeynhausen, Germany

**Keywords:** heart failure, ventricular assist device, telemonitoring, implantable cardioverter defibrillator, cardiac resynchronization therapy, outcome

## Abstract

The telemonitoring of heart failure (HF) patients is becoming increasingly important. This study aimed to evaluate the benefit of telemonitoring in end-stage HF patients with a ventricular-assistance device (VAD). A total of 26 HF-patients (66 ± 11 years, 88% male) on VAD therapy with an implantable cardioverter-defibrillator (ICD) or a cardiac resynchronization defibrillator (CRT-D) including telemonitoring function were enrolled. The long-term follow-up data (4.10 ± 2.58 years) were assessed. All the patients (*n* = 26, 100%) received daily ICD/CRT-D telemonitoring. In most of the patients (73%, *n* = 19), the telemedical center had to take action for a mean of three times. An acute alert due to sustained ventricular arrhythmias (VAs) occurred in 12 patients (63%) with 50% of them (*n* = 6) requiring ICD shock delivery. Eight patients (67%) were hospitalized due to symptomatic VAs. In 11 patients (92%), immediate medication adjustments were recommended. Relevant lead issues were revealed in thirteen patients (50%), with six patients (46%) undergoing consecutive lead revisions. Most of the events (83%) were detected within 24 h. Daily telemonitoring significantly reduced the number of in-hospital device controls by 44% (*p* < 0.01). The telemonitoring ensured that cardiac arrhythmias and device/lead problems were identified early, allowing pre-emptive and prompt interventions. In addition, the telemonitoring significantly reduced the number of in-hospital device controls in this cohort of HF patients.

## 1. Introduction

In patients with end-stage heart failure (HF), the implantation of a ventricular assist device (VAD) offers a therapeutic option in bridge to heart transplant (HTx) [1,2]. Patients with advanced HF often suffer from ventricular arrhythmias (Vas), requiring a pharmacological or an interventional electrophysiological treatment [3,4,5,6]. Thus, most HF patients benefit from an implantable cardioverter-defibrillator (ICD) or cardiac resynchronization defibrillator (CRT-D) in addition to HF consensus medication prior to VAD implantation [3,6]. 

The telemonitoring of HF patients is a rapidly evolving field. ICDs and CRT-Ds with a telemonitoring function afford the chance to routinely monitor physiological and technical data [7,8,9,10]. Important predisposing factors for poor clinical outcomes in HF-patients include ventricular arrhythmias (VAs), defibrillator shocks, the onset of atrial fibrillation (AF), a low percentage of biventricular pacing (BiV) and abnormal lead parameters [7,8,9,10,11,12,13,14,15,16]. Accompanying outpatient care, the multiparametric telemonitoring of HF patients leads to reduced hospitalization rates, fewer days in hospital due to cardiac decompensations and significantly reduced mortality among these patients [11,12,13,15,16]. Daily device-based (ICD, CRT-D) telemonitoring allows for a reduction in in-hospital follow-up visits, leading to improved patient compliance and reduced workloads for clinical staff [17]. In addition, daily telemonitoring offers the possibility of the early detection of VAs, as well as device or lead problems, which is associated with increased patient safety [14], reduced hospitalization rates and mortality [13,15,16]. However, the benefits of a daily device-based multiparameter-telemonitoring in this specific cohort of patients with an implantable VAD for end-stage HF have not yet been evaluated. 

## 2. Methods

In this single-center observational study, 26 end-stage HF-patients with an implantable VAD and daily device-based (ICD, CRT-D) multiparametric telemonitoring at our institute for applied telemedicine (IFAT) were included. Patients were included consecutively and not because of prior device-related problems or frequent cardiac arrhythmias. Long-term follow-up data were assessed retrospectively. The study was performed in compliance with the principles outlined in the Declaration of Helsinki and approved by the Institutional Ethics Committee (reg. no. 2022-994). 

## 3. Data Collection

Data on patient characteristics, medication, symptoms, complications, device programming, arrhythmias, hospitalization rates and mortality were compiled from patient records and discharge letters. Procedural parameters and clinical aspects were taken from surgery reports and procedure-related documents.

## 4. Statistical Analysis

All statistical analyses were performed with SPSS, version 27 (SPSS, Inc., Chicago, IL, USA). Continuous variables between the groups (telemonitoring vs. no telemonitoring) were compared by employing an unpaired two-sided Student’s *t*-test. Categorial data were examined by Fisher’s exact test. Data are presented as mean ± SD or percentage value unless stated otherwise. A *p*-value < 0.05 was considered statistically significant.

## 5. Results

### 5.1. Patient Characteristics

The study population consisted of 26 consecutive end-stage HF patients (66 ± 11 years, 88% male) with an implantable VAD and daily device-based multiparameter-telemonitoring. Most of the patients received VAD implantation as destination therapy (DT) (*n* = 22, 85%) with four patients awaiting HTx in bridge to transplant (BTT) (15%) indication. In all the patients (*n* = 26, 100%), ICD- or CRT-D-devices had been implanted for primary prevention, on average, 3.73 years prior to VAD therapy. All the patient characteristics are summarized in Table 1.

### 5.2. ICD-/CRT-D-Programming

In total, 42% of the patients (*n* = 11) had an ICD, while 58% (*n* = 15) had a CRT-D implant. After VAD implantation, anti-tachycardia therapy was deactivated in two patients (8%). During the observation period, VA-zone reprogramming was documented in 12 patients (46%). Two further patients underwent device reprogramming due to lead problems (8%). Although 20 patients (77%) had a two-chamber device, 54% (*n* = 14) were programmed in VVI mode. One patient (4%) underwent a downgrade of his CRT-D-device. Further details are presented in Table 2. 

### 5.3. VAD-Related Complications 

Ventricular-assist-device-related complications were reported in 73% of the patients (*n* = 19). On average, two hospital admissions for severe VAD complications were required in 54% of the patients (*n* = 14). The time to the first VAD complication amounted to 239 ± 113 days. The following complications were documented: driveline infections (*n* = 18, 69%), gastrointestinal bleeding (*n* = 7, 27%), deranged INR values (*n* = 4, 15%), VAD thrombosis (*n* = 2, 8%), intracerebral bleeding (*n* = 1, 4%) and stroke (*n* = 1, 4%). 

### 5.4. Follow-up Data

The long-term follow-up data (4.10 ± 2.58 years) were assessed.

### 5.5. Patient Outcomes 

**Figure 1:**
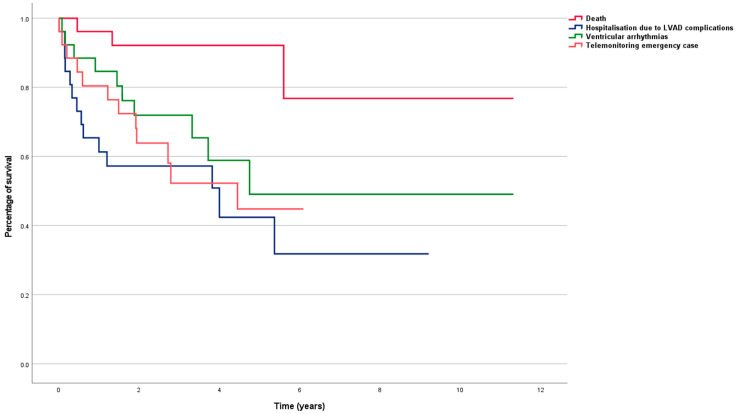
Kaplan–Meier plot on event-free survival.

Recurrent overnight admissions to hospital were reported in 21 patients (81%) for the following reasons: worsening of heart failure (*n* = 7, 27%), VAs (*n* = 8, 31%) and VAD complications (*n* = 14, 54%). During the observation period, three patients (12%) died following VAD complications, which included sepsis following severe driveline infection (*n* = 2, 8%) and acute intracerebral bleeding (*n* = 1, 4%). One patient received HTx (4%). Twelve patients (46%) suffered from sustained VAs. The telemedical center had to take action for 73% of the patients (*n* = 19). The event-free survival rates were visualized through Kaplan–Meier-plot analyses (Figure 1).

During the observation period, three patients (12%) died due to VAD complications. Hospital admissions for severe VAD complications were required in 54% of the patients (*n* = 14). Ventricular arrhythmias were documented in 12 patients (63%). Telemonitoring alerts requiring immediate action were documented in 63% of the study population (*n* = 12). 

### 5.6. Impact of Telemonitoring on Patient Treatment and Safety

**Figure 2:**
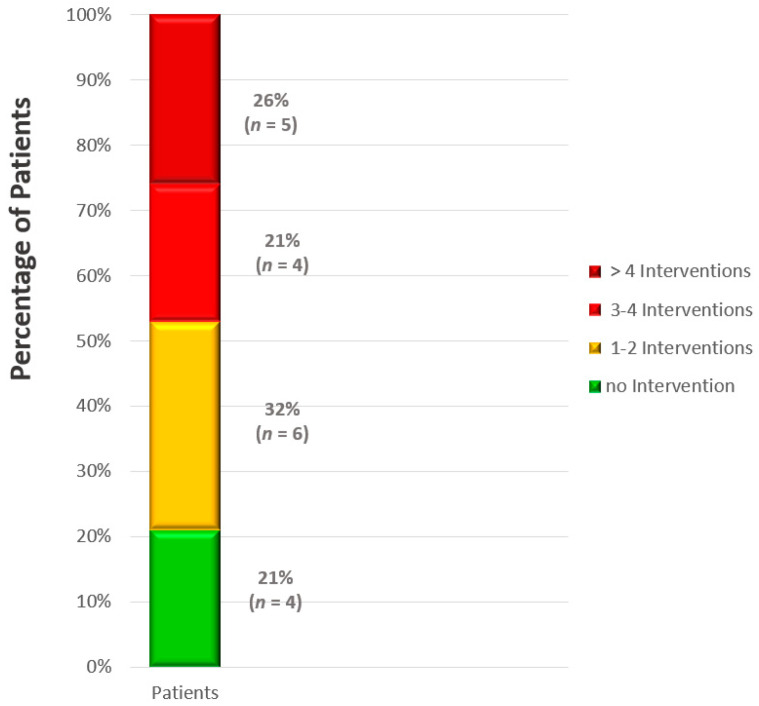
Frequency of interventions relating to telemonitoring alerts.

All the patients (*n* = 26, 100%) with a VAD-device implant for end-stage HF received daily telemedical ICD or CRT monitoring. On average, three interventions by the telemedical center were required for the majority of the patients (*n* = 19, 73%). The frequency of interventions per patient is depicted in Figure 2. Twelve patients (63%) presented in an emergency requiring immediate action on the day of alarm identification. 

In 19 patients (73%) the telemedical center had to intervene due to telemonitoring alerts. In four patients (21%), no events occurred; in six patients (32%), action was required 1–2 times; in four patients (21%), action was required 3–4 times; and in 5 patients (26%), action was required more than four times.

### 5.7. Device and Lead-Related Complications

**Figure 3:**
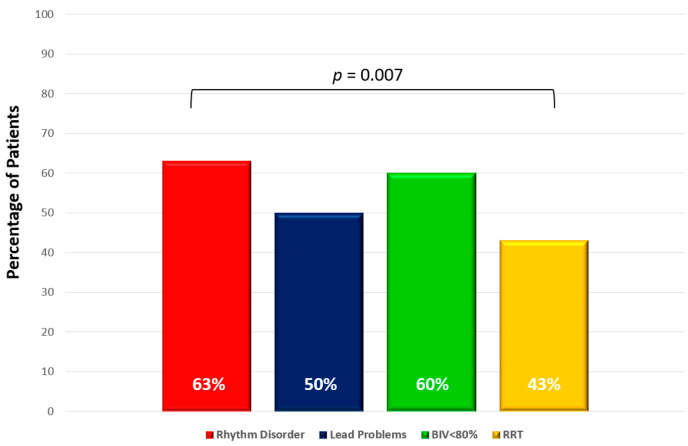
Distribution of telemonitoring alerts.

Telemonitoring revealed ICD or CRT-D related complications in 14 patients (54%). Six of them (43%) were scheduled for device replacements due to a recommended replacement time (RRT)-alert. Lead problems were documented in thirteen patients (50%) with six of them (46%) undergoing consecutive lead revision. In eight patients (62%), the transmitted IEGMs revealed the first signs of lead dysfunction. Five patients (38%) were identified through abnormal lead parameters. During the study’s observation period, immediate hospitalization for a right ventricular (RV)-lead revision was initiated in two patients (15%). The details are summarized in Figure 3.

An acute alert due to sustained VAs was documented in 12 patients (63%). Lead problems were documented in thirteen patients (50%), with six of them (46%) undergoing consecutive lead revisions. In 60% of the patients (*n* = 9) with a CRT-D-device (*n* = 15), the percentage of BiV had fallen below 80% due to recurrent VAs and a high proportion of PVCs. Six patients (43%) were scheduled for device replacements due to a RRT-alert.

### 5.8. Biventricular Pacing (BiV)

In 60% of the patients (*n* = 9) with a CRT-D-device (*n* = 15), the percentage of BiV decreased below 80% due to recurrent VAs and a high proportion of premature ventricular contractions (PVC) (Figure 3). In most of the patients (78%, *n* = 7), prompt medication adjustments (e.g., beta-blockers and antiarrhythmic agents) led to an improved biventricular pacing rate >80%. 

### 5.9. Cardiac Arrhythmias

An acute alert for sustained VAs was documented in 12 patients (63%). All the sustained VAs were treated with anti-tachycardia pacing (ATPs). Of these, 58% (*n* = 7) required an additional shock delivery. Inadequate shocks occurred in 25% of the patients (*n* = 3). Eight patients (67%) were transferred to hospital by the telemedical center due to symptomatic VAs and/or shock delivery. In 11 patients (58%), immediate medication adjustments (e.g., beta-blocker and antiarrhythmic agents) were recommended. Two patients (17%) underwent consecutive VA-ablation procedures for recurrent, symptomatic medication-refractory VAs. The distribution of the telemonitoring alerts is summarized in Table 3. 

### 5.10. Telemonitoring Events and Time to Detection

**Figure 4:**
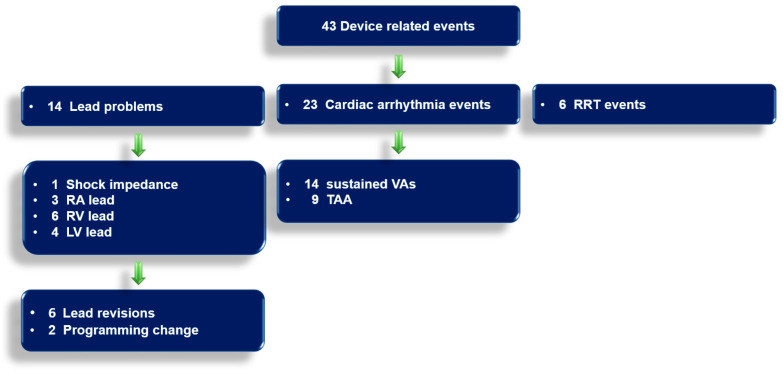
Telemonitoring Events. RA, right atrium; RV, right ventricle; LV, left ventricle; VAs, ventricular arrhythmias; TAA, tachyarrhythmia absoluta; RRT, recommended replacement time.

The telemonitoring revealed 43 device-related events. A total of 14 lead-problem notifications (33%) comprising out-of-range atrial and ventricular lead impedance and sensing, as well as out-of-range shock impedance, were observed, requiring six lead revisions (43%) and two reprogramming changes (14%). Twenty-three cardiac arrhythmias (53%) occurred in the study cohort of patients. The details are presented in Figure 4. 

**Figure 5:**
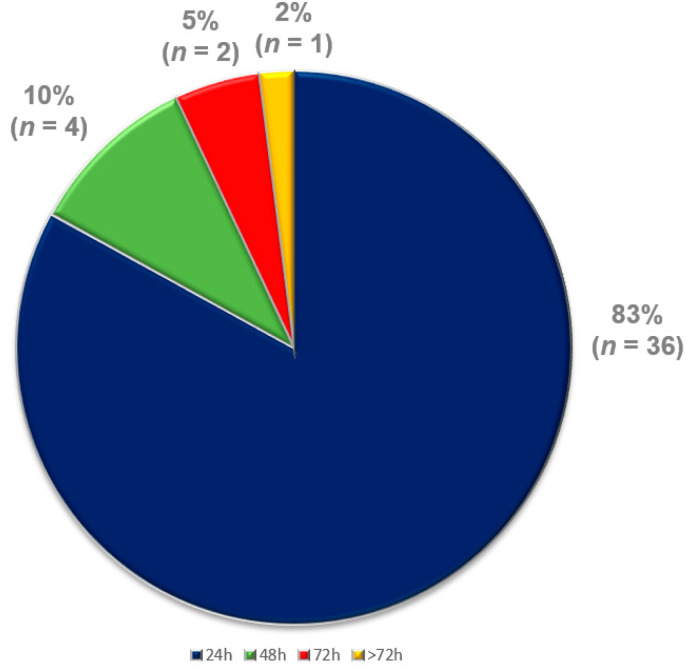
Days to detection of events.

Concerning the duration to detection, thirty-six events (83%) were detected within 24 h, four (10%) were detected 24–48 h later and only three events (7%) were detected more than 48 h later (Figure 5). 

Thirty-six events (83%) were detected within 24 h, four (10%) were detected 24–48 h later and only three events (7%) were detected more than 48 h later. 

### 5.11. Effects of Telemonitoring on In-Hospital Device Controls 

As part of routine clinical practice, patients without telemedical care are scheduled for in-hospital device controls every 6 months, whereas patients included in device-telemonitoring programs undergo in-hospital controls only once a year.

**Figure 6:**
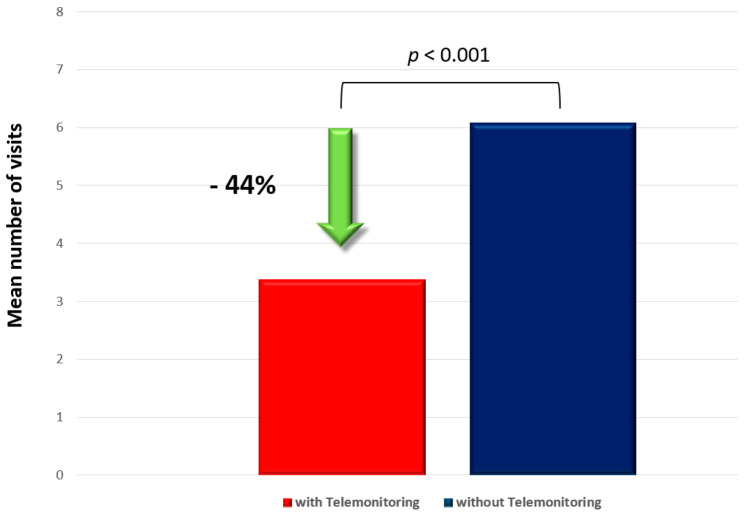
Patients’ in-hospital device controls.

In contrast to a hypothetical control group of patients without telemedical care with in-hospital device controls every 6 months, the patients included in the device-telemonitoring program underwent in-hospital controls only once a year. Although 11 additional in-hospital device controls were scheduled because of clinically relevant telemonitoring alerts, significantly fewer in-hospital device-controls were scheduled in the telemonitoring cohort of patients compared to the patients without telemedical care (Telemonitoring: 3.64 ± 2.02 controls; No-Telemonitoring: 6.16 ± 3.55; *p* < 0.01). Thus, daily device telemonitoring significantly reduced the absolute number of in-hospital visits, by 44% (*p* < 0.01) (Figure 6).

The telemonitoring significantly reduced the number of in-hospital follow-up device controls in the VAD patients (*p* < 0.001). 

## 6. Discussion and Main Findings

This is the first study to evaluate daily device-based multiparametric telemonitoring in end-stage HF patients with a VAD-device implant in terms of benefits, complication rates and outcomes, providing the longest follow-up in a study of this extent so far. 

This study has three major findings: 

Daily implant-based multiparameter-telemonitoring in VAD patients is feasible and efficient.

Device telemonitoring may increase patient safety as it allows pre-emptive interventions and the immediate detection of cardiac arrhythmias and device/lead problems.

Even in the specific cohort of end-stage HF-patients, telemonitoring may significantly reduce the number of in-hospital device controls.

### 6.1. Telemonitoring in HF Patients

#### 6.1.1. Benefits in End-Stage HF

In prior studies, all the patients with an ICD capable of telemetry [14,17] or HF patients with a reduced ejection fraction (EF) <35% and HF symptoms (New York Heart Association (NYHA) functional class I–III) for more than 3 months and an ICD or CRT-D with telemonitoring function were included [13,15,16]. As reported in the IN-TIME study, routine, daily, implant-based, multiparametric telemonitoring is feasible and significantly improves outcomes for patients with HF [15]. As presented in a sub-analysis of the IN-TIME study, HF patients with a CRT-D, who are at higher risk of worse outcomes compared to HF patients with an ICD only, present with a higher absolute benefit from telemonitoring [16]. Therefore, the data suggest particularly beneficial effects of telemonitoring for high-risk patients. This is consistent with our results, as we demonstrated that telemonitoring is feasible and efficient even in end-stage HF-patients with an implantable VAD (Table 1, Table 2 and Table 3, Figure 1, Figure 2, Figure 3, Figure 4, Figure 5 and Figure 6). As reported in the In-Time study, the favorable effect of telemonitoring seems to arise from careful attention to various kinds of telemonitoring data without a single typical scenario [15]. Compared to prior studies, our study provides the longest follow-up (4.10 ± 2.58 years) so far [13,14,15,16].

#### 6.1.2. Impact on Time to Detection of Events and Patient Safety

Although systemic circulation is supported by the VAD, the occurrence of sustained VAs in VAD patients may have a significant impact on morbidity and mortality [18]. Implant-based telemonitoring may play a decisive role in handling clinically relevant cardiac arrhythmias and device/lead trouble, through immediate identification (Table 2 and Table 3, Figure 1, Figure 3, Figure 4 and Figure 5). This is of special importance as most of the events were not recognized by the patients themselves, as verified by their telephone calls. Thus, relevant issues would have only been detected in the case of a severe subsequent device dysfunction or at the next routine in-hospital check-up. Hence, immediate follow-up appointments with physicians or necessary medication adjustments would not have been made without telemedical observations.

A prior study analyzing the same-day discovery rate of ICD dysfunctions, even in asymptomatic patients, reported an overall detection rate of 51% within 24 h [14]. We present an even higher identification rate, of 83%, for all cardiac arrhythmias and device dysfunctions (*n* = 36) within 24 h (Figure 5). This tool may allow pre-emptive or early interventions, e.g., medication adjustments, device reprogramming and hospital admissions, including device/lead revisions, as well as VA-ablation procedures (Table 2 and Table 3, Figure 4). Prior studies, reporting on the feasibility, safety and efficacy of catheter ablations for VAs in third-generation VAD patients [5,19] and for AT/AF in end-stage HF patients strengthen these therapeutic options through rapid identification [20]. 

The documented delay in telemonitoring-data transmission is primarily due to the fact that not all devices transmit events directly. A transmitter is required and patients have to be close to the transmitter for the data to be transmitted. Furthermore, the telemonitoring program analyzed is not an emergency program, which means that device platforms are not monitored 24/7 by telehealth nurses and physicians. Nevertheless, far better and earlier event detection is possible in telemonitoring patients compared to patients without telemedical care, as some HF patients either do not notice clinically relevant changes or, despite the appearance of symptoms, do not contact a physician. Thus, although the same-day discovery of device and lead issues is affected by engineering differences, transmission frequency, methods of alert notification and workflow patterns [14], telemonitoring greatly increases patient safety.

#### 6.1.3. Impact on Pre-Emptive Interventions

Apart from the early detection of the onset or progression of cardiac arrhythmias, the telemonitoring of patients may benefit from the early recognition of suboptimal device function. 

A 100% biventricular pacing rate is highly desirable in CRT-D patients as it is associated with left-ventricular remodeling and reduced mortality rates [21]. In 60% of patients (*n* = 9), among whom the percentage of BiV had fallen below 80% (Figure 3), the telemonitoring allowed early recognition and pre-emptive interventions to quickly increase the BiV stimulation rate again. Furthermore, the telemonitoring allowed the early detection of lead issues. Thus, it may protect patients against inappropriate shocks. 

#### 6.1.4. Impact on In-Hospital Device Controls 

One prior study reported a significant reduction in face-to face visits, without a significant increase in unscheduled-follow ups in pacemaker and ICD patients with implant-based telemonitoring [17]. In contrast to our study, most of the patients included in this study did not suffer from HF. Nevertheless, these findings are consistent with our results as we revealed that, even in the specific cohort of end-stage HF-patients, telemonitoring may significantly reduce the number of in-hospital device controls (Figure 6).

### 6.2. ICD Programming in VAD Patients 

Currently, the role of ICD therapy in VAD patients remains unclear [2]. Although ICDs may be effective in terminating VA episodes, patients often experience shocks while conscious [2]. In some VAD patients, a more conservative approach, accompanied by a VA-monitoring strategy using telemonitoring alerts, may be safe [22]. To reduce the number of shocks in primarily asymptomatic VAD patients, 12 patients (46%) underwent an adjustment of tachytherapy zones after VAD implantation (Table 2). In other VAD patients, who are at high risk of VAs and hemodynamic collapse, an ICD may provide benefits [23]. For this reason, the reprogramming of ICD therapy after VAD implantation was evaluated carefully and individualized for each patient (Table 2). Telemedical surveillance allowed the immediate detection of cardiac arrhythmias in the entire cohort of patients (Figure 4 and Figure 5). 

## 7. Future Perspectives and Clinical Outlook

The future establishment of a complete VAD-telemonitoring program, including ICD-, CRT-D, INR and VAD care, may be highly beneficial. A prospective randomized controlled trial analyzing the role of ICD therapy in VAD patients is warranted.

## 8. Conclusions

Daily implant-based multiparametric telemonitoring in VAD patients is feasible and efficient. It may increase patient safety as it allows the early detection of cardiac arrhythmias and device/lead issues. In addition, telemonitoring significantly reduces the number of in-hospital follow-up device controls in this specific high-risk cohort of HF-patients. Further studies are warranted to confirm these initial observations.

## 9. Limitations

Because of the small patient population, the lack of a control group and the retrospective design of our study, we report initial observations. Future studies are required to confirm our results. 

## Figures and Tables

**Table 1:** Patient Characteristics.

Characteristics	Patients (*n* = 26)
Age (years)	66 ± 11
Gender, male	23 (88%)
BMI (kg/m^2^)	28 ± 6
LVEF (%)	22 ± 4
Years since VAD-implantation	4.10 ± 2.58
VAD (DT)	22 (85%)
VAD (BTT)	4 (15%)
NYHA class III	26 (100%)
ICM	15 (58%)
DCM	12 (46%)
Stroke/TIA	12 (46%)
Hypertension	23 (88%)
Diabetes mellitus	11 (42%)
AT/AF	21 (81%)
CKD	22 (85%)
BB	26 (100%)
Amiodarone	22 (85%)
Phenprocoumon	26 (100%)
AV-node ablation	3 (12%)

Continuous variables are shown as the mean ± SD and categorical variables as the number (%). BMI, body-mass index; LVEF, left ventricular ejection fraction; VAD, ventricular-assistance device; DT, destination therapy; BTT, bridge to transplant; NYHA class, New York Heart Association class; ICM, ischemic cardiomyopathy; DCM, dilated cardiomyopathy; TIA, transient ischemic attack; AT/AF, atrial tachycardia/atrial fibrillation; CKD, chronic kidney disease; BB, beta blocker.

**Table 2:** ICD-/CRT-D Programming.

Characteristics	Patients (*n* = 26)
One-chamber ICD	6 (23%)
Two-chamber ICD	5 (19%)
CRT-D	15 (58%)
Years since device implantation	7.83 ± 4.01
RVp (%)	67 ± 42
VVI mode	14 (54%)
AT/AF burden (%)	25 ± 12
PVC burden (%)	17 ± 5
VT/VF therapy activated	24 (92%)
VT/VF therapy (ATP + shock)	10 (38%)
VT(ATP only)/ VF therapy	11 (42%)
VF therapy only	3 (13%)
Tachycardia-zone reprogramming	12 (46%)
Lead-problem reprogramming	2 (8%)
Device downgrading	1 (4%)

Continuous variables are shown as the mean ± SD and categorical variables as the number (%). ICD, implantable cardioverter defibrillator; CRT-D; cardiac-resynchronization-therapy defibrillator; RVp, right ventricular pacing; AT/AF, atrial tachycardia/atrial fibrillation; PVC, premature ventricular contraction; ATP, antitachycardia pacing; VT, ventricular tachycardia; VF, ventricular fibrillation.

**Table 3:** Patients with Cardiac Arrhythmia Alerts.

Characteristics	Patients
Sustained VAs	12 (63%)
ATP therapy	12 (100%)
Adequate shock delivery	7 (58%)
AT/AF	3 (12%)
Inadequate shock delivery	3 (25%)
Hospital admission	8 (67%)
Medication adjustment	11 (92%)
Scheduled for VA-ablation	2 (17%)

Categorical variables are shown as the number (%). VA, ventricular arrhythmias; ATP, antitachycardia pacing; AT/AF, atrial tachycardia/ atrial fibrillation.

## Data Availability

The data underlying this article will be shared on reasonable request to the corresponding author.
